# Structural Aspects of Potential Endocrine-Disrupting Activity of Stereoisomers for a Common Pesticide Permethrin against Androgen Receptor

**DOI:** 10.3390/biology10020143

**Published:** 2021-02-11

**Authors:** Ishfaq Ahmad Sheikh, Mohd Amin Beg

**Affiliations:** 1 King Fahd Medical Research Center, King Abdulaziz University, Jeddah 21589, Saudi Arabia; mbeg@kau.edu.sa; 2 Department of Medical Laboratory Technology, Faculty of Applied Medical Sciences, King Abdulaziz University, Jeddah 21589, Saudi Arabia

**Keywords:** androgen receptor, chirality, endocrine disruption, permethrin, stereoisomers, reproductive dysfunction

## Abstract

**Simple Summary:**

Human exposure to synthetic or naturally occurring endocrine-disrupting compounds (EDCs) contaminating the environment is associated with disruption in endocrine signaling and homeostatic imbalance of hormones. Pyrethroids constitute an important class of extensively used insecticides reported to have endocrine-disrupting activity. Permethrin is one of the most commonly used pyrethroids and exists in isomeric forms. The aim of this study was to investigate and compare the potential endocrine-disrupting activity of permethrin isomers against the androgen receptor (AR). Structural binding studies showed that all permethrin isomer compounds have the potential to compete with native ligand binding in the AR ligand binding pocket. In conclusion, the results of this study suggest that human exposure to commercially produced isomeric forms of permethrin could potentially interfere with the AR function, which may lead to male reproductive dysfunction.

**Abstract:**

Endocrine-disrupting chemicals (EDCs) are a serious global public health and environmental concern. Pyrethroids are insecticide chemicals that are extensively used for crop protection and household purposes but have been identified as EDCs. On account of their ubiquitous environmental presence, human exposure occurs via food, dermal, or inhalation routes and is associated with health problems, including reproductive dysfunction. Permethrin is the most commonly used pyrethroid, and with two chiral centers in its structure, it has four stereoisomeric forms (two enantiomer pairs), i.e., permethrin (1*R*,3*R*)-*cis*, permethrin (1*R*,3*S*)-*trans*, permethrin (1*S*,3*S*)-*cis*, and permethrin (1*S*,3*R*)-*trans*. The current study was performed for predicting the potential endocrine-disrupting activity of the aforementioned four stereoisomers of permethrin against the androgen receptor (AR). The structural binding characterization and binding energy estimations in the AR binding pocket were done using induced fit docking. The structural binding data indicated that all stereoisomers were placed stably in the AR binding pocket and that the estimated binding energy values were comparable to the AR native ligand, except for permethrin (1*S*,3*S*)-*cis*. Furthermore, the commonality in the amino acid interactions to that of the AR native ligand and the binding energy values suggested the potential AR-disrupting activity of all the stereoisomers; however, stereoselective differences were not observed. Taken together, the results suggest that human exposure to permethrin, either as a racemate mixture or in individual stereoisomer form, could potentially interfere with AR function, which may lead to male reproductive dysfunction.

## 1. Introduction

Endocrine-disrupting chemicals (EDCs) are synthetic or natural compounds that interfere with hormone systems in humans and animals, causing harmful impacts on their health [1,2]. Pyrethroids, an important class of insecticides, are extensively used in the agriculture industry for crop protection and as household insecticides but are identified as EDCs by the United States Environmental Protection Agency due to their hormone-like activity [3,4,5,6]. Pyrethroids are synthetic structural analogs of pyrethrin, which is a natural insecticide derived from *Chrysanthemum cinerariaefolium* flowers [7,8]. The pyrethroid insecticides became popular due to their low application rate, longer half-life, and lower environmental concerns compared to other classes of insecticides and were further boosted by the phase-out of organochlorine and organophosphorus pesticides. Global pyrethroid usage accounted for about 35% of total insecticides and was worth US$3.2 billion in 2019 [9,10] with projections to reach $3.8 billion by 2027 [11]. Even though the environmental persistence of pyrethroids is very low (less than 90 days) [12], they are ubiquitously present in the environment due to their high volume and consistent agricultural and household applications [13]. The abundant environmental levels of pyrethroids have ensured high human exposure, and thus, pyrethroids have emerged as an important global health concern. In this regard, various studies have reported pyrethroids and their metabolites from human samples [14,15,16,17].

A significant number of studies have associated pyrethroid exposure in humans with endocrine-disrupting effects on the hypothalamic–pituitary–gonadal (HPG) axis (reviewed in [3]). For example, frequently detected pyrethroids along with their metabolites have shown antagonistic activity against the human androgen receptor (AR) in a luciferase reporter gene assay [18]. Studies on male laboratory rodents have shown a significant increase in serum levels of follicle-stimulating hormone and luteinizing hormone on exposure to different types of pyrethroids [19,20,21,22]. Furthermore, studies have also shown that pyrethroids negatively impacted serum testosterone levels, Leydig cells, sperm DNA, sperm quality, and sperm count in male rats, resulting in fertility problems [23,24]. 

The majority of the pyrethroid compounds contain a cyclopropane ring in their structure as most of them are derivatives of 2, 2-dimethylcyclopropanecarboxylic acid [25]. However, these compounds show chirality due to the presence of two chiral centers in their cyclopropane ring, hence generating stereoisomers. The stereoisomers often demonstrate differential toxicological and insecticidal activities [26,27,28]. Enantiomer-specific toxicological profiling has been an emerging area of research and has been receiving overwhelming attention in the scientific community. Permethrin is one of the very commonly used pyrethroids with a cyclopropane ring in its structure; four stereoisomers (two enantiomeric pairs), i.e., permethrin (1*R*3*R*)-*cis*, permethrin (1*R*3*S*)-t*rans*, permethrin (1*S*3*S*)-*cis,* and permethrin (1*S*3*R*)-*trans*, have been reported for permethrin [29,30,31]. Limited studies on the potential endocrine-disrupting activity of enantiomers of permethrin are available, especially related to the reproductive system. One of the possible mechanisms for endocrine disruption of pyrethroid insecticides could be through disruption of AR ligand binding. The AR belongs to the steroid nuclear receptor protein family and binds to androgens, primarily testosterone and dihydrotestosterone, which are vital for male reproductive physiology and development [32]. The EDCs have been reported to interfere in the interactions of AR with its native ligand and may result in adverse effects on AR function, resulting in homeostatic imbalance and/or signaling of sex steroid hormones [33,34,35]. 

Studies on enantiomer-specific permethrin docking simulation with the AR have not been reported to the best of our knowledge. Therefore, the present study was done to investigate the potential disruptive effects of permethrin stereoisomers against the AR with the aim of elucidating the structural binding pattern and molecular interactions of the four permethrin enantiomers, permethrin (1*R*3*R*)-*cis*, permethrin (1*R*3*S*)-*trans*, permethrin (1*S*3*S*)-*cis,* and permethrin (1*S*3*R*)-*trans*, with the AR. This study was expected to provide insight into the potential enantioselective endocrine-disrupting role of permethrin that may lead to dysfunction in male reproductive function and development.

## 2. Materials and Methods

The commonly used pyrethroid compound, permethrin, was chosen for the present study. The three-dimensional structure of permethrin was downloaded from the PubChem compound database (https://pubchem.ncbi.nlm.nih.gov/; accessed on 20 December 2020). All four possible stereoisomers were generated from the given structure and were subjected to structural binding studies using the Schrodinger 2017 suite with Maestro 11.4 as a graphical user interface (Schrodinger, LLC, New York, NY, USA, 2017). The detailed methodology was already described in our previous study [36]. 

### 2.1. Protein Preparation

The coordinates for the three-dimensional crystal structure of the AR in complex with its native ligand, testosterone, solved at 1.64 Å resolution (PDB code: 2AM9), was retrieved from the Protein Data Bank (PDB; http://www.rcsb.org/; accessed on 20 December 2020). The downloaded crystal complex was subjected to further processing and prepared for docking studies using the Protein Preparation Wizard workflow of Schrodinger Glide (Schrodinger suite 2017-4; Schrodinger, LLC) as described in our previous study [36]. Briefly, during the protein preparation step, we firstly imported the crystal complex structure of the AR to the Glide docking software. Then, hydrogen atoms and charges were added, and water molecules were also removed. This was followed by hydrogen bond network optimization and energy minimization. 

### 2.2. Ligand Preparation

**Figure 1:**
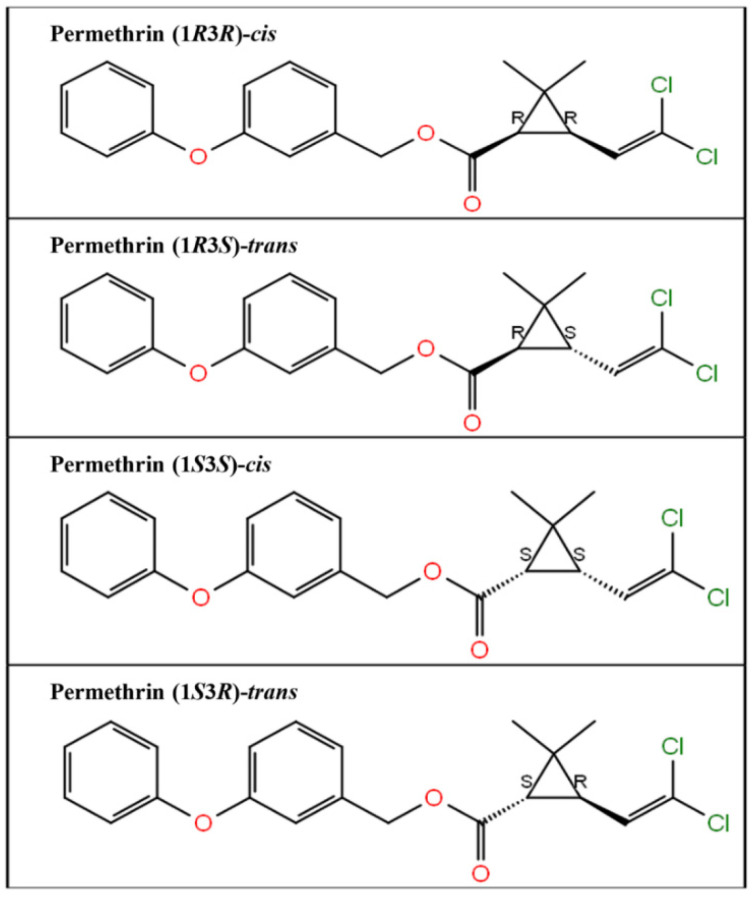
Two-dimensional structure of the four stereoisomers of permethrin, i.e., permethrin (1*R*3*R*)-*cis*, permethrin (1*R*3*S*)-*trans*, permethrin (1*S*3*S*)-*cis*, and permethrin (1*S*3*R*)-*trans*.

The three-dimensional structure of the ligand compound, permethrin, was downloaded from the PubChem compound database. The PubChem compound identity of permethrin is 40326. The ligand compound was prepared for docking studies using the LigPrep module (Schrodinger 2017: LigPrep, Schrodinger, LLC). LigPrep generated four permethrin stereoisomers, permethrin (1*R*3*R*)-*cis*, permethrin (1*R*3*S*)-*trans*, permethrin (1*S*3*S*)-*cis*, and permethrin (1*S*3*R*)-*trans.* The two-dimensional structures of all four generated stereoisomers are presented in Figure 1.

### 2.3. Induced Fit Docking

The Schrodinger’s Induced Fit Docking (IFD) module was employed for the docking of all four stereoisomers of permethrin in the ligand binding site of the AR. The detailed IFD methodology was described previously [36]. The grid was generated at the native ligand testosterone-binding site to perform the IFD of all four permethrin stereoisomer ligands. In IFD, the flexibility is induced both in the ligand binding pocket of the protein receptor as well as in the ligand. The IFD protocol was developed and validated by the Schrodinger-based Glide and Refinement module in Prime to accurately predict the ligand binding poses and the associated changes in the receptor ligand binding pocket. Firstly, the receptor was subjected to constrained minimization during the protein preparation step with an RMSD cutoff of 0.18 Å. Then, for each ligand, initial Glide docking was performed using a softened potential and optional side chain removal. The maximum number of poses retained by default per ligand was 20. Then, for each receptor–ligand complex, side chains were predicted using Prime for amino acid residues falling within a 5 Å distance for any ligand pose and was followed by minimization. In addition, the ligand was also minimized for each complex (receptor–ligand) pose. At this point, the structure of the receptor in each pose was the reflection of the induced fit for the ligand conformation and structure. It was followed by Glide re-docking within a specified energy of the lowest-energy structure of every receptor–ligand complex structure. It was finally followed by IFD score estimations for each pose. The identical general plan was followed in the extended sampling protocol. The native ligand, testosterone, was also subjected to IFD in the ligand-binding pocket of the AR. 

### 2.4. Binding Affinity Calculations

The ligand binding affinity estimation for all four stereoisomers of permethrin against the AR was performed by employing the Prime module of Schrodinger 2017 with the MMGB-SA function as described previously [36].

## 3. Results

**Figure 2:**
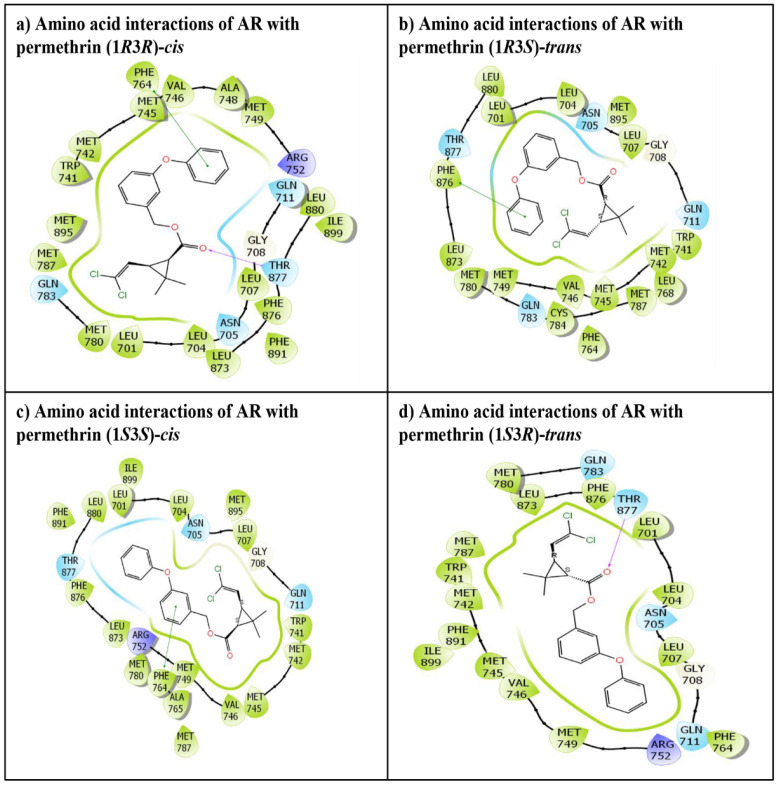
The amino acid residue interactions of the four stereoisomers of permethrin in the ligand binding pocket of the AR: (**a**) permethrin (1*R*3*R*)-*cis*, (**b**) permethrin (1*R*3*S*)-*trans*, (**c**) permethrin (1*S*3*S*)-*cis*, and (**d**) permethrin (1*S*3*R*)-*trans*. The cyan colored amino acids lining the ligand binding pocket represent polar residues. The green colored amino acid residues represent hydrophobic residues, and the purple colored amino acids represent positively charged residues. The red colored arrows indicate hydrogen bonding interactions, and the green colored lines indicate pi–pi interaction between the ligand and receptor amino acid residues.

**Figure 3:**
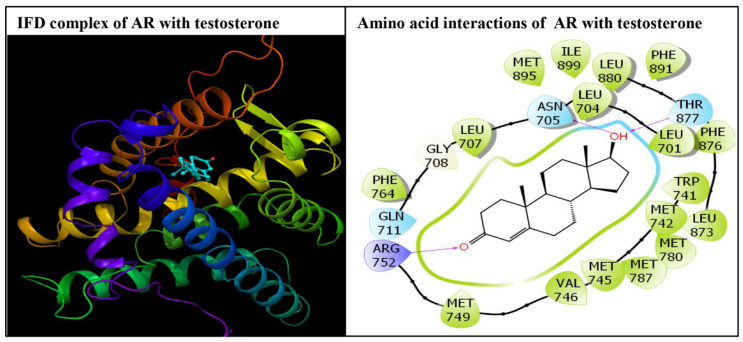
The docking display of the androgen receptor (AR) in complex with the native ligand, testosterone (left panel), and the molecular interactions of testosterone with residues lining the AR ligand binding pocket (right panel). The cyan colored amino acids lining the ligand binding pocket represent polar residues. The green colored amino acid residues represent hydrophobic residues, and the purple colored amino acid represents positively charged residues. The red colored arrows indicate hydrogen bonding interactions between the ligand and receptor amino acid residues.

The IFD of all four stereoisomers of permethrin, permethrin (1*R*3*R*)-*cis*, permethrin (1*R*3*S*)-*trans*, permethrin (1*S*3*S*)-*cis*, and permethrin (1*S*3*R*)-*trans*, was successfully executed in the ligand binding pocket of the AR. All the ligands were placed tightly, indicating the stable binding of these ligands. The IFD experiment generated numerous docking poses for each ligand, but only the best (highest-ranking) poses were chosen and considered for advanced molecular interaction analysis. These poses, depicting various amino acid interactions for each stereoisomer as well as the native ligand, testosterone, are presented in Figure 2 and Figure 3. The docking complexes of all four permethrin stereoisomers demonstrated that 21–24 amino acid residues in the AR ligand binding pocket were involved in various molecular interactions (Figure 2).

The native ligand, testosterone, docking display exhibited interactions with 22 amino acid residues in the AR ligand binding pocket (Figure 3).

Two cis-stereoisomers, permethrin (1*S*3*S*)-*cis* (Figure 2a) and permethrin (1*R*3*R*)-*cis* (Figure 2c), interacted with 23 and 24 amino acid residues, respectively, in the AR ligand binding pocket. Overall, 100% commonality in interacting AR amino acid residues was observed between the native ligand, testosterone, and both of the aforementioned *cis*-enantiomers. Both cis-enantiomers displayed one pi–pi interaction with the amino acid residue Phe-764. In addition, the permethrin (1*R*3*R*)-*cis* enantiomer also displayed one hydrogen binding interaction with the Thr-877 residue. The remaining two stereoisomers, permethrin (1*R*3*S*)-*trans* (Figure 2b) and permethrin (1*S*3*R*)-*trans* (Figure 2d) (*trans*-enantiomers), displayed interactions with 22 and 21 amino acid residues of the AR, respectively. However, approximately 86% commonality was observed in the interacting residues of the native ligand, testosterone, and each trans-enantiomer. Further, permethrin (1*R*3*S*)-*trans* displayed one pi–pi interaction with Phe-876, and permethrin (1*S*3*R*)-*trans* displayed hydrogen bonding interaction with Thr-877.

IFD-associated parameters such as IFD score, Glide score, dock score, and binding energy values for all four stereoisomers of permethrin as well as for the AR native ligand, testosterone, are presented (Table 1). The IFD scores, Glide scores, and dock scores of all the stereoisomers were similar to the values calculated for the native ligand, testosterone. Furthermore, the binding energy values of all ligands except permethrin (1*S*3*S*)-*cis* were comparable to the native ligand. The binding energy values calculated for permethrin (1*S*3*S*)-*cis* were lower than the values calculated for the other three stereoisomers.

## 4. Discussion

The aim of the present study was to investigate and characterize the structural binding interactions of the four stereoisomers of permethrin, i.e., permethrin (1*R*3*R*)-*cis*, permethrin (1*R*3*S*)-*trans*, permethrin (1*S*3*S*)-*cis*, and permethrin (1*S*3*R*)-*trans*, against the AR in order to gain information on their potential endocrine-disrupting properties. Permethrin gains access to the human body by either dietary or non-dietary sources and has been reported as a potential disruptor of hormone homeostasis [37,38,39]. The IFD data revealed the stable placement and tight binding of all the indicated stereoisomers of permethrin in the AR binding pocket. Furthermore, the amino acid interactions and other parameters, such as docking score, Glide score, etc., of these ligands were similar to the native ligand, testosterone. The estimated binding energy values were also comparable to the native ligand except for one enantiomer, i.e., permethrin (1*S*3*S*)-*cis*, which was lower than the native ligand, testosterone. All these data indicate the tight binding of these stereoisomers in the AR pocket. In summary, the overall commonality in molecular docking pattern, amino acid interactions, and other parameters between permethrin stereoisomers and the AR native ligand, testosterone, indicate that these permethrin stereoisomers have the potential to interfere in AR binding. This potential interference may lead to the disruption of AR signaling and result in abnormal male reproductive function and development. 

The reported declining trends in the testosterone levels and semen quality of men across the globe have been thought to be partly due to human exposure to EDCs including pyrethroid pesticides [40,41,42]. The last two decades have seen an exponential increase in the use of pyrethroid pesticides on account of them being considered relatively safe longer-acting derivatives of natural pyrethrins and because of the withdrawal of more toxic organophosphorus insecticides from commercial applications [43]. In this regard, permethrin was recently [44] shown to be one of the two most detected pyrethroids compounds in the environment and the metabolites of permethrin, i.e., 3-phenoxybenzoic acid (3-PBA) and *cis*- and *trans*-3-2,2-dichlorovinyl)-2,2-dimethylcyclopropane carboxylic acid (cis-DCCA and trans-DCCA) have been frequently detected in human fluids. In addition, permethrin was reported in environmental media such as surface water, soils, sediments, indoor houses, crops, aquatic organisms, land organisms, and human samples from 33 countries about 73 times, with the highest concentration (800.00 mg/kg) and frequency of detection (65.0%) reported in residential environments. China and the United States were the two top countries that produced and extensively used permethrin [44]. Hence, due to its ubiquitous distribution and large-scale use, initially, permethrin was included in the list of chemicals for screening under the United States Environmental Protection Agency (USEPA)-administered Endocrine Disruptor Screening Program [45]. Biomonitoring of pyrethroid metabolites, including those of permethrin (3-PBA, cis-DCCA, and trans-DCCA), in the general public has been done in many countries. Permethrin metabolites have been detected in the general population, including children, in the USA, France, and Japan with a detection frequency of 90–100% in the general population [46,47,48]. In the USA, the average intake of permethrin by an adult is estimated to be about 3.2 µg/day [49]. According to WHO guidelines, permethrin levels in drinking water should not exceed 20 µg/L. The Occupational Safety and Health Administration of the United States has set the occupational exposure limits for an 8-h workday, 40-h workweek to be 5 mg/m³ [49]. Recently, the USEPA classified permethrin as “likely to be carcinogenic to humans” [50]. 

Our current study on the structural binding interactions of permethrin with the AR was an attempt to predict the AR disruption potential of the four stereoisomers of permethrin. Previous molecular docking studies of permethrin with sex steroid receptors, especially the human AR, including studies on enantiomer-specific permethrin docking, are not available. However, in one study [51], the structural binding of permethrin with zebrafish transthyretin (a circulatory thyroid-hormone transport protein) was shown to form effective van der Waals interactions with six key amino acid residues. The study suggested that the binding of permethrin with transthyretin and interference with its activity contributed, partly, to the thyroid endocrine disruption of permethrin in fish; the permethrin isomer used in the docking study was not indicated. The reported study provided support for our results of the binding of all the stereoisomers of permethrin with the AR, which was comparable to the binding of the native ligand testosterone. Reported in vitro binding studies for permethrin with the AR have been equivocal, with a strong antagonistic activity of permethrin against the AR using a reporter gene expression assay in one study [18]. Another radio-ligand based study reported that permethrin interacted competitively with the AR [52]. In contrast, poor antiandrogenic activity of permethrin was shown in another similar study [53] using a CV-1 cell line reporter gene expression assay. The reasons for the differing results of these studies are not known. 

Our results on the potential interference of permethrin against the AR are also supported by epidemiological studies. Epidemiological studies on infertile men showed that urinary concentrations of permethrin metabolites 3-PBA, cis-DCCA, and trans-DCCA were positively correlated with FSH and LH concentrations, while trans-DCCA was negatively correlated with estradiol, testosterone, and the free androgen index [42,54]. Further antiandrogenic support comes from a number of studies showing a negative relationship between urinary 3-PBA levels and the sperm concentration and quality parameters, such as motility, morphology, sperm DNA damage, aneuploidy rates, and y:x ratio, of men [43,55]. However, studies showing no association of urinary 3-PBA levels and serum hormone concentrations have also been reported [43]. Nevertheless, it is generally agreed that pyrethroids, including permethrin, negatively impact reproduction in men. 

Further, studies in laboratory animals have also suggested antiandrogenic effects, such as reduced sperm motility and sperm count, decreased testes weight, reduced levels of testosterone, structural abnormalities in testes and decreased libido [22,24,56,57,58,59]. Of particular interest and relevance to the current research are various studies that have reported the stereoselective effects of permethrin [28,60]. In this regard, the stereoselective estrogenic activity of permethrin was reported to be due to stereoselectivity in the biotransformation of permethrin into metabolites with more estrogenic activity [28]. A study in rats given oral doses of mixtures of *cis*-permethrin and *trans*-permethrin reported higher levels of *cis*-permethrin than *trans*-permethrin in all organs [61], suggesting differences in absorption and cellular uptake among the permethrin stereoisomers. Moreover, permethrin was reported to show stereoselectivity in esterase and oxidase metabolism in human liver microsomes [62]. A study on rat adrenal pheochromocytoma (PC12) cells showed the stereoselective induction of oxidative stress and cytotoxicity by permethrin [60]. In addition, in embryo-larval zebrafish, significant differences were observed among the stereoisomers in inducing estrogen-responsive gene expression [63]. 

Up to the present time, there has been no insecticide discovered or synthesized that is toxic to insects but safe for humans. Although pyrethroids, including permethrin, are thought to have low toxicity to humans, children may be more vulnerable because of higher exposure through breast milk and house dust and lower metabolism and higher accumulation in their tissues [64]. Therefore, caution is necessary while using insecticide chemicals. An urgent concern arises about the unknown adverse health effects of long-term exposure as well as the latent effects of permethrin. Long-term reproductive studies involving transgenerational and epigenomic experiments in laboratory animals and epidemiological studies in both low- and high-risk population groups are suggested.

## 5. Conclusions 

The current study was performed to ascertain the potential stereoselective endocrine-disrupting role of permethrin by gaining insights into the structural binding of its four stereoisomers, permethrin (1*R*3*R*)-*cis*, permethrin (1*R*3*S*)-*trans*, permethrin (1*S*3*S*)-*cis*, and permethrin (1*S*3*R*)-*trans*, in the ligand binding pocket of the AR. All the aforementioned stereoisomers showed very tight binding with the AR, and the estimated binding energy values were close to the native ligand, testosterone, except for permethrin (1*S*3*S*)-***cis***. Although the data for their binding patterns and the estimated binding energy values for the four stereoisomers did not indicate any stereoselective differences, the close binding energy and high commonality of all the amino acid interactions to that of the native ligand, testosterone, suggest the potential AR-disrupting activity of all permethrin stereoisomers. Taken together, the results of this study suggest that human exposure to permethrin, either as a racemate mixture or in individual stereoisomer form, could potentially interfere with AR function, which may lead to male reproductive dysfunction. 

## Figures and Tables

**Table 1:** Molecular docking parameters and binding energy values of four stereoisomers of permethrin, i.e., permethrin (1*R*3*R*)-*cis*, permethrin (1*R*3*S*)-*trans*, permethrin (1*S*3*S*)-*cis*, and permethrin (1*S*3*R*)-*trans*, and the androgen receptor (AR) native ligand, testosterone.

Ligand	Number of Interacting AR Residues	Percentage of Interacting Residues Common with Native Ligand (%)	IFD Score	Docking Score (Kcal/mol)	Glide Score (Kcal/mol)	MMGB-SA (Kcal/mol)
Permethrin (1*R*3*R*)-*cis*	24	100	−575.06	−10.87	−10.87	−145.62
Permethrin (1*R*3*S*)-*trans*	22	86	−575.58	−10.57	−10.57	−144.54
Permethrin (1*S*3*S*)-cis	23	100	−574.66	−10.37	−10.37	−134.02
Permethrin (1*S*3*R*)-*trans*	21	86	−575.70	−10.42	−10.42	−144.76
Testosterone	22	100	−577.54	−12.85	−12.87	−152.82

## Data Availability

All data is contained within the article.
